# Supplementary material for: Correlated insulator collapse due to quantum avalanche via in-gap ladder states
Source: Nat Commun. 2023 May 22;14:2936. doi: 10.1038/s41467-023-38557-8 (PMC10203265; doi:10.1038/s41467-023-38557-8)
Supplement: Supplementary file 1 — Supplementary Information [file 41467_2023_38557_MOESM1_ESM.pdf]

SUPPLEMENTARY INFORMATION

Correlated Insulator Collapse due to Quantum Avalanche via In-Gap Ladder States

Jong E. Han,[1,*] Camille Aron,[2] Xi Chen,[1] Ishiaka Mansaray,[1] Jae-Ho Han,[3] Ki-Seok Kim,[4] Michael Randle,[5] and Jonathan P. Bird[1,5]

[1]*Department of Physics, State University of New York at Buffalo, Buffalo, New York 14260, USA*

[2]*Laboratoire de Physique de l'École Normale Supérieure, ENS, Université PSL, CNRS, Sorbonne Université, Université Paris Cité, F-75005 Paris, France*

[3]*Center for Theoretical Physics of Complex Systems, Institute for Basic Science(IBS), Daejeon 34126, South Korea*

[4]*Department of Physics, POSTECH, Pohang, Gyeongbuk 37673, South Korea*

[5]*Department of Electrical Engineering, State University of New York at Buffalo, Buffalo, New York 14260, USA*

* jonghan@buffalo.edu

S1. Theory: Model and Green's Function Formalism

Model: The Hamiltonian consists of electron and phonon parts as [1]

$$H = H_{\rm el} + H_{\rm ph} + H_{\rm ep} + H_{\rm diss}, \tag{S1}$$

with the electron part $H_{\rm el}$ modeled on a tight-binding lattice with electrostatic potential $V(\mathbf{r}) = -e\mathbf{E}\cdot\mathbf{r}$ on the site $\mathbf{r}$ of an infinite honeycomb lattice, the phonon part $H_{\rm ph}$ with Einstein phonons of energy $\hbar\omega_{\rm ph}$, the electron-phonon (el-ph) coupling $H_{\rm ep}$ and the dissipation mechanism for both electrons and phonons being contained in $H_{\rm diss}$. The electron part is given as Eq. (8) in the main text. We suppressed the summation over the electron spin and later take spin degeneracy into account in the self-energies. The phonon part of the Hamiltonian is also defined in the main text.

The dissipation $H_{\rm diss}$ has two parts, for electrons and phonons, respectively, as $H_{\rm diss} = H_{\rm diss,el} + H_{\rm diss,ph}$. The dissipation into fermion baths can be understood as particle exchange between hot and cold electrons in the sample and substrate, respectively. We model the fermion bath as a continuum of fermion states, with each state being labeled as k, coupled to each site independently [1] as $H_{\rm diss,el} = \sum_{\mathbf{r}} H_{\rm diss,el}(\mathbf{r})$ with

$$H_{\rm diss,el}(\mathbf{r}) = \sum_\alpha \sum_k \left[(\epsilon_k - \mathbf{E}\cdot\mathbf{r})\, c^\dagger_{\alpha\mathbf{r}}(k) c_{\alpha\mathbf{r}}(k) + \frac{\gamma}{\sqrt{N}} d^\dagger_{\alpha\mathbf{r}} c_{\alpha\mathbf{r}}(k) + \text{H.c.} \right]. \tag{S2}$$

Here, $c^\dagger_{\alpha\mathbf{r}}(k)$ ($c_{\alpha\mathbf{r}}(k)$) is the creation (annihilation) operator of bath fermions of the orbital α and the continuum index k defined at each site $\mathbf{r}$, ϵ_k the dispersion relation, γ the overlap to the main orbital $d^\dagger_{\alpha\mathbf{r}}$ ($d_{\alpha\mathbf{r}}$), and N the normalization factor against the length of the bath chain. We assume a structureless model for the density of states of infinite bandwidth with the energy-independent hybridization Γ as

$$\frac{\gamma^2}{N} \sum_k \delta(\omega - \epsilon_k) \equiv \Gamma. \tag{S3}$$

The dissipation of the phonons can be formulated similarly, now with coupling to an Ohmic bath at each site. With $H_{\rm diss,ph} = \sum_{\mathbf{r}} H_{\rm diss,ph}(\mathbf{r})$

$$H_{\rm diss,ph}(\mathbf{r}) = \sum_k \left[\frac{1}{2}\left(q^2_{\alpha\mathbf{r}}(k) + \nu_k^2 x^2_{\alpha\mathbf{r}}(k) \right) + \frac{\alpha_k}{\sqrt{N}} x_{\alpha\mathbf{r}}(k) \varphi_{\alpha\mathbf{r}} + \text{H.c.} \right]. \tag{S4}$$

Here, $x_{\alpha\mathbf{r}}(k)$ is the amplitude of the bath particle with the continuum index k of orbital α and $q_{\alpha\mathbf{r}}(k)$ is its conjugate momentum, ν_k is the dispersion relation, and α_k is the overlap to the phonon field $\varphi_{\alpha\mathbf{r}}$ on the main lattice. In the Ohmic limit, we define the decay rate of the phonons into the bath to be

$$\frac{1}{N} \sum_k \frac{\alpha_k^2}{\nu_k^2} \delta(\omega - \nu_k) \equiv \tau_P^{-1} \omega, \tag{S5}$$

with the phonon life-time parameter τ_P.

Green's function technique: Within the Keldysh Green's function technique, the dissipative bath can be easily incorporated in the (non-interacting) self-energy as

$$\Sigma^R_{0,\alpha\beta}(\mathbf{r},\omega) = -i\Gamma\delta_{\alpha\beta} \text{ and } \Sigma^<_{0,\alpha\beta}(\mathbf{r},\omega) = 2i\Gamma f_0(\omega + \mathbf{E}\cdot\mathbf{r})\delta_{\alpha\beta}, \tag{S6}$$

for the retarded and lesser Green's functions for electrons, respectively. $f_0(\omega) = [e^{\omega/k_BT} + 1]^{-1}$ is the Fermi-Dirac function at the bath temperature T (k_B = Boltzmann constant). Phonon dissipation is similarly described within the dissipative phonon self-energy as

$$\Pi^R_0(\omega) = -2i\tau_P^{-1}\omega \text{ and } \Pi^<_0(\omega) = -4i\tau_P^{-1}\omega n_0(\omega), \tag{S7}$$

with the Bose-Einstein function $n_0(\omega) = [e^{\omega/k_BT} - 1]^{-1}$. Note that the electric field does not couple directly to the charge-neutral phonons and the phonon Green's functions are translationally invariant. As suggested in the self-energy expression, the electron Green's functions in the steady-state limit satisfy the symmetry relation

$$G^{R,<}_{\alpha\beta}(\mathbf{r} + a\hat{\mathbf{x}},\omega) = G^{R,<}_{\alpha\beta}(\mathbf{r},\omega + Ea), \tag{S8}$$

which embodies the symmetry requirement that, other than the energy shift relative to the electrostatic energy and chemical potential shift, the electronic properties such as electron occupation number are translationally invariant.

We solve the electron-phonon problem by mutually consistent interacting self-energies to electrons and phonons through iteration. The approximation is that we consider the perturbation up to second-order of the el-ph coupling $g_{\rm ep}$, and we make the self-energies as diagonal in site and orbital. The electron self-energy by the el-ph coupling is given as

$$\Sigma^{\lessgtr}_{\mathrm{ep},\alpha}(\mathbf{r},\omega) = ig^2_{\rm ep}\int \frac{\mathrm{d}\omega'}{2\pi}\mathcal{G}^{\lessgtr}_{\alpha\alpha}(\mathbf{r},\omega-\omega')D^{\lessgtr}_{\alpha}(\omega'), \tag{S9}$$

where $\mathcal{G}^{\lessgtr}(\mathbf{r},\omega)$ is the electronic *impurity* (Weiss field) Green's function in the DMFT formalism [3, 4] and $D^{\lessgtr}(\omega)$ phonon's on-site Green's function. The phonon self-energy is similarly defined as

$$\Pi^{\lessgtr}_{\mathrm{ep},\alpha}(\omega) = -2ig^2_{\rm ep}\int \frac{\mathrm{d}\omega'}{2\pi}G^{\lessgtr}_{\alpha\alpha}(\mathbf{r},\omega+\omega')G^{\gtrless}_{\alpha\alpha}(\mathbf{r},\omega'), \tag{S10}$$

with the full electron Green's function $G^{\lessgtr}(\mathbf{r},\omega)$. The pre-factor 2 is due to spin degeneracy. The retarded and advanced self-energies are computed according to the standard definition [4]. Here, we use the convention that the phonon Green's function is defined with respect to the field variable, eg., $D^>_\alpha(t) = -i\langle\varphi_{\alpha\mathbf{r}}(t)\varphi_{\alpha\mathbf{r}}(0)\rangle$.

Once the local self-energies are computed, we construct the full Green's function for each perpendicular wave-vector p_y as described in Refs. [1, 2]. Once the local full Green's functions are computed, we calculate the Weiss-field Green's function $\mathcal{G}(\omega)$ to perform the DMFT self-consistency. Due to the broken spatial symmetry, the equilibrium version with the momentum summation is not applicable. The modified Dyson's equation becomes for the site $\mathbf{r} = 0$,

$$\mathcal{G}^R(\omega) = [I + G^R(\omega)\Sigma^R_{\rm ep}(\omega)]^{-1}G^R(\omega), \tag{S11}$$

$$\mathcal{G}^<(\omega) = [I + G^R(\omega)\Sigma^R_{\rm ep}(\omega)]^{-1} G^<(\omega)[I + \Sigma^A_{\rm ep}(\omega)G^A(\omega)]^{-1} - \mathcal{G}^R(\omega)\Sigma^<_{\rm ep}(\omega)\mathcal{G}^A(\omega), \quad \text{(S12)}$$

where the product involves matrix multiplication over orbital indices. The impurity and the full Green's functions are then iterated with the self-energies, Eqs. (S9,S10), until convergence is reached.

The phonons do not need the impurity Green's functions since their self-energy only consists of the electron Green's functions. Moreover, the electric-field does not couple directly to phonons and phonons are not subject to the condition Eq. (S8). Therefore, the equilibrium-like momentum summation is valid for phonons. For Einstein phonons,

$$D^R(\omega) = \left[\omega^2 - \omega_0^2 + 2i\omega/\tau_P - \Pi^R_{\rm ep}(\omega)\right]^{-1}$$
$$D^<(\omega) = (\Pi^<_{\rm ep}(\omega) - 4i\omega n_0(\omega)/\tau_P)|D^R(\omega)|^2, \quad \text{(S13)}$$

with the Bose-Einstein function $n_0(\omega)$ at the bath temperature. With the acoustic phonons, the integral over the phonon spectra is done analytically with the dispersion relation and the phonon density of states, respectively, as

$$\omega_k = sk \text{ with } s = \omega_D/(6\pi^2)^{1/3}, \text{ and } \nu_{\rm ph}(\omega) = \frac{\omega^2}{2\pi^2 s^3}\Theta(\omega_D - \omega). \quad \text{(S14)}$$

Then the phonons Green's functions become

$$D^R(\omega) = \int \frac{\nu_{\rm ph}(\epsilon)}{\omega^2 - \epsilon^2 + 2i\omega/\tau_P - \Pi^R_{\rm ep}(\omega)} {\rm d}\epsilon = \frac{-\omega_D + f}{2\pi^2 s^3} \quad \text{(S15)}$$
$$D^<(\omega) = \int \frac{\left[\Pi^<_{\rm ep}(\omega) - 4i\omega n_0(\omega)\right]\nu_{\rm ph}(\epsilon)}{|\omega^2 - \epsilon^2 + 2i\omega/\tau_P - \Pi^R_{\rm ep}(\omega)|^2} {\rm d}\epsilon = \frac{-\left[\Pi^<_{\rm ep}(\omega) - 4i\omega n_0(\omega)\right](f - f^*)}{(2\pi^2 s^3)(z - z^*)}$$

with

$$z = \omega^2 + 2i\omega/\tau_P - \Pi^R_{\rm ep}(\omega) \text{ and } f = \sqrt{-z}\tan^{-1}\left(\frac{\omega_D}{\sqrt{-z}}\right). \quad \text{(S16)}$$

The local electronic spectral function per orbital is defined as

$$A_\alpha(\omega) = -\frac{1}{\pi}{\rm Im}G^R_{\alpha\alpha}(\mathbf{r} = 0, \omega), \quad \text{(S17)}$$

and the local distribution functions for electron and phonon, respectively, as

$$f(\omega) = -\frac{1}{2}\frac{\sum_\alpha {\rm Im}G^<_{\alpha\alpha}(\mathbf{r} = 0, \omega)}{\sum_\alpha {\rm Im}G^R_{\alpha\alpha}(\mathbf{r} = 0, \omega)}, \quad n(\omega) = \frac{1}{2}\frac{{\rm Im}D^<(\omega)}{{\rm Im}D^R(\omega)}. \quad \text{(S18)}$$

As shown in Fig. 3 of the main text, the orbital-summed Green's function $\sum_\alpha G^<_{\alpha\alpha}(\mathbf{r} = 0, \omega)$ is particle-hole symmetric. Since these distribution functions reduce to the equilibrium Fermi-Dirac and Bose-Einstein functions at the $E = 0$ limit, respectively, we extend the electron and phonon effective temperatures as

$$T^2_{\rm el} = \frac{6}{\pi^2}\int_{-\infty}^{\infty} \omega[f(\omega) - \Theta(-\omega)]{\rm d}\omega$$
$$T^2_{\rm ph} = \frac{6}{\pi^2}\int_0^{\infty} \omega n(\omega){\rm d}\omega, \quad \text{(S19)}$$

with the step-function $\Theta(x)$. As shown in Fig. 2(b) in the main text, the electronic distribution function may deviate strongly from the Fermi-Dirac form. In such situations, the above definition for the effective temeprature serves as a approximate measure of nonequilibrium excitations.

Parametrization of the electron-phonon coupling: We briefly comment on the parametrization of the electron-phonon coupling by the mass-renormalization factor λ. We use the commonly used definition for λ as

$$\lambda = \frac{1}{\Omega} \sum_k \frac{g_{\rm ep}^2}{\omega_k^2} \nu_0, \tag{S20}$$

with the density of electronic states ν_0 (per site and per spin) replaced by the inverse bandwidth of a $2D$ lattice as $\nu_0 = (8t)^{-1}$. Ω is the area of the Brillouin zone. For the optical phonon at frequency $\omega_{\rm ph}$, the mass-renormalization λ becomes

$$\lambda_{\rm optical} = \frac{1}{8t} \left(\frac{g_{\rm ep}}{\omega_{\rm ph}} \right)^2, \tag{S21}$$

and for the acoustic phonon with the Debye frequency ω_D [see Eq. (S14)],

$$\lambda_{\rm acoustic} = (8t)^{-1} \int_0^{\omega_D} \left(\frac{g_{\rm ep}}{\omega} \right)^2 \nu_{\rm ph}(\omega) {\rm d}\omega = \frac{3}{8t} \left(\frac{g_{\rm ep}}{\omega_D} \right)^2. \tag{S22}$$

Dependence of avalanche with model parameters: Here we evaluate the validity of the heuristic derivation for the avalanche condition [Eq. (2) in the main text]

$$E_{\rm av} \sim \frac{\hbar \Gamma \Delta}{e g_{\rm ep}^2} \left[\frac{(\hbar \omega_{\rm ph})^3}{2m} \right]^{1/2}. \tag{S23}$$

We test this schematic relation in the regime of small avalanche field by comparing with fully numerical calculations. Fig. S1 show a Log-Log plot of $E_{\rm av}$ as a function of our model parameters (damping rate from the fermion bath Γ, energy gap Δ, phonon-frequency $\omega_{\rm ph}$ and electron-phonon coupling constant $g_{\rm ep}$). As detailed in the caption, $E_{\rm av}$ and Γ, Δ, $\omega_{\rm ph}^{3/2}$ and $g_{\rm ep}^{-2}$ are normalized by their minimum values so all curves start at $(1, 1)$. The above equation predicts that $E_{\rm av}$ is an increasing function of the variables shown on the horizontal axis, which is numerically verified without any doubt. The curves are clustered around the linear relation (black-dashed line) and, except for the Γ dependence, the relations slowly roll over to a sub-linear dependence.

The linear dependence on Γ is not only highly nontrivial, as discussed in the main text, but also numerically robust for a wide range of parameter sets. The departure to the slight super-linear behavior can be understood as a result of interaction-generated electron-dephasing at high fields. As $E_{\rm av}$ increases, the electron self-energy develops stronger dephasing from the electron-phonon coupling and adds to the bare dephasing rate Γ, which then means that higher electric field are needed to cause the avalanche.

The relation $E_{\rm av} \propto g_{\rm ep}^{-2}$ confirms the scenario that el-ph coupling triggers an avalanche. The dependence on $\omega_{\rm ph}$ further corroborates the role of the electron-phonon

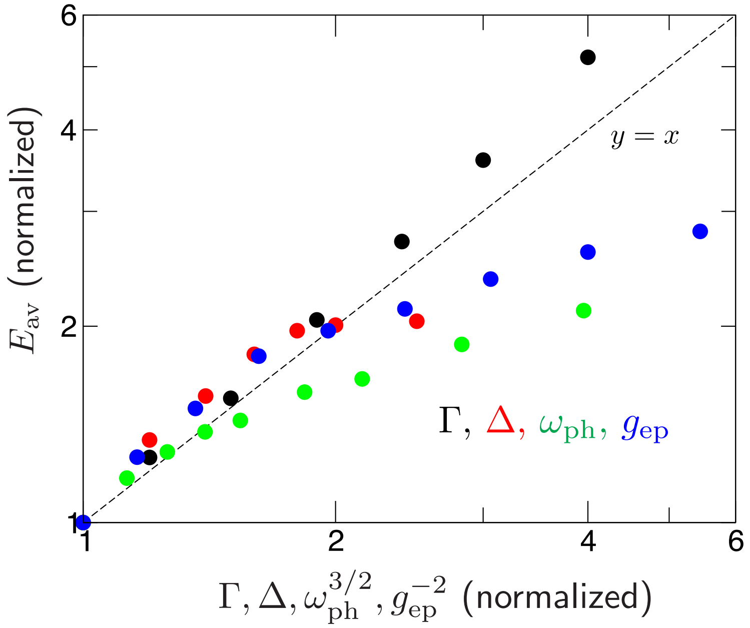

Fig. S1. Log-Log plot for the avalnche fields $E_{\rm av}$ versus the fermion damping Γ, the energy gap Δ, 3/2-power of the phonon frequency $\omega_{\rm ph}^{3/2}$, and the squared-inverse of the electron-phonon coupling, $g_{\rm ep}^{-2}$. The parameters are normalized so that each set of data starts at $(1, 1)$. The Γ-run (black) has other parameters of $\Delta = 1$, $\omega_{\rm ph} = 0.3$, $g_{\rm ep} = 0.5$ with $E_{\rm av,min} = 0.00089$ at $\Gamma_{\rm min} = 0.001$. For the Δ-run (red), $\Gamma = 0.005$, $\omega_{\rm ph} = 0.3$, $g_{\rm ep} = 0.7$ with $E_{\rm av,min} = 0.00254$ at $\Delta_{\rm min} = 0.5$. For the $\omega_{\rm ph}$-run (green), $\Gamma = 0.005$, $\Delta = 1$, $g_{\rm ep} = 0.6$ with $E_{\rm av,min} = 0.00265$ at $\omega_{\rm ph,min} = 0.12$. For the $g_{\rm ep}^{-2}$-run (blue), $\Gamma = 0.005$, $\Delta = 0.5$, $\omega_{\rm ph} = 0.3$ with $E_{\rm av,min} = 0.00254$ at $g_{\rm ep,max} = 0.7$. Throughout all runs, the phonon life-time is $\tau_P = 1000$ and the bath temperature $T_b = 0.001$. For comparison, curve of exponent 1 (black-dashed) is shown.

coupling. While we used the commonly quoted expression $(g_{\rm ep}/\omega_{\rm ph})^2$ for the strength of the electron-phonon coupling's contribution to the electron self-energy [*eg.* see Eq. (S21)] in deriving Eq. (2), it is only a rough estimate and the resulting exponent 3/2 to $\omega_{\rm ph}$ should not be taken too literally. We note the general tendency towards sub-linear relations for $E_{\rm av}$ vs. Δ, $\omega_{\rm ph}^{3/2}$ and $g_{\rm el}^{-2}$ at higher $E_{\rm av}$. This results from nonperturbative effects in the self-consistent solution. As we approach the regime that requires higher avalanche field, higher order interaction effectively enhances electron-phonon coupling, causing the avalanche to occur at smaller fields.

Based on these analyses, we conclude that Eq. (2) derived in the main text is conceptually sound and captures the essential physics that the avalanche results from the competition between electron dephasing and electron-phonon generated replica states.

REFERENCES

[1] Li J, Han JE. Nonequilibrium excitations and transport of Dirac electrons in electric-field-driven graphene. Phys. Rev. B **97**, 205412 (2018).

[2] Li, J, Aron, C, Kotliar, G & Han, JE, Electric-Field-Driven Resistive Switching in the Dissipative Hubbard Model. Phys. Rev. Lett. **114**, 226403 (2015).

[3] Georges, A., Kotliar, G., Krauth, W. & Rozenberg, M. Dynamical mean-field theory of strongly correlated fermion systems and the limit of infinite dimensions. Rev. Mod. Phys. **68**, 13 (1996).

[4] Aoki, H. et al. Nonequilibrium dynamical mean-field theory and its applications. Rev. Mod. Phys. **86**, 779 (2014).

[5] Nathawat, J. et al. Signatures of hot carriers and hot phonons in the re-entrant metallic and semiconducting states of Moiré-gapped graphene. Nat. Comm. **14**, 1507 (2023).
